# T Cells Plead for Rejuvenation and Amplification; With the Brain’s Neurotransmitters and Neuropeptides We Can Make It Happen

**DOI:** 10.3389/fimmu.2021.617658

**Published:** 2021-03-22

**Authors:** Mia Levite

**Affiliations:** ^1^ Faculty of Medicine, The Hebrew University, Jerusalem, Israel; ^2^ Institute of Gene Therapy, Hadassah University Hospital, Jerusalem, Israel

**Keywords:** T cells, Neurotransmitters, Neuropeptides, Dopamine, Glutamate, Nerve-Driven Immunity, Immunotherapy, Adoptive Cell Therapy

## Abstract

T cells are essential for eradicating microorganisms and cancer and for tissue repair, have a pro-cognitive role in the brain, and limit Central Nervous System (CNS) inflammation and damage upon injury and infection. However, in aging, chronic infections, acute SARS-CoV-2 infection, cancer, chronic stress, depression and major injury/trauma, T cells are often scarce, exhausted, senescent, impaired/biased and dysfunctional. People with impaired/dysfunctional T cells are at high risk of infections, cancer, other diseases, and eventually mortality, and become multi-level burden on other people, organizations and societies. It is suggested that “Nerve-Driven Immunity” and “Personalized Adoptive Neuro-Immunotherapy” may overcome this problem. Natural Neurotransmitters and Neuropeptides: Glutamate, Dopamine, GnRH-II, CGRP, Neuropeptide Y, Somatostatin and others, bind their well-characterized receptors expressed on the cell surface of naïve/resting T cells and induce multiple direct, beneficial, and therapeutically relevant effects. These Neurotransmitters and Neuropeptides can induce/increase: gene expression, cytokine secretion, integrin-mediated adhesion, chemotactic migration, extravasation, proliferation, and killing of cancer. Moreover, we recently found that some of these Neurotransmitters and Neuropeptides also induce rapid and profound decrease of PD-1 in human T cells. By inducing these beneficial effects in naïve/resting T cells at different times after binding their receptors (*i.e*. NOT by single effect/mechanism/pathway), these Neurotransmitters and Neuropeptides by themselves can activate, rejuvenate, and improve T cells. “Personalized Adaptive Neuro-Immunotherapy” is a novel method for rejuvenating and improving T cells safely and potently by Neurotransmitters and Neuropeptides, consisting of personalized diagnostic and therapeutic protocols. The patient’s scarce and/or dysfunctional T cells are activated *ex vivo* once by pre-selected Neurotransmitters and/or Neuropeptides, tested, and re-inoculated to the patient’s body. Neuro-Immunotherapy can be actionable and repeated whenever needed, and allows other treatments. This adoptive Neuro-Immunotherapy calls for testing its safety and efficacy in clinical trials.

## The Enormous Problem: T Cells are Scarce, Severely Impaired, and Dysfunctional in Numerous Human Beings, Leading to Harsh Multi-Level Consequences and Implications

T cells are essential for eradicating infectious organisms and cancer, immune response to injury, organ repair, and for various other health-promoting missions (1). T cells have also various beneficial and important roles in the healthy brain, where they have pro-cognitive as well as neuroprotective roles (2, 3).

**Figure 1:**
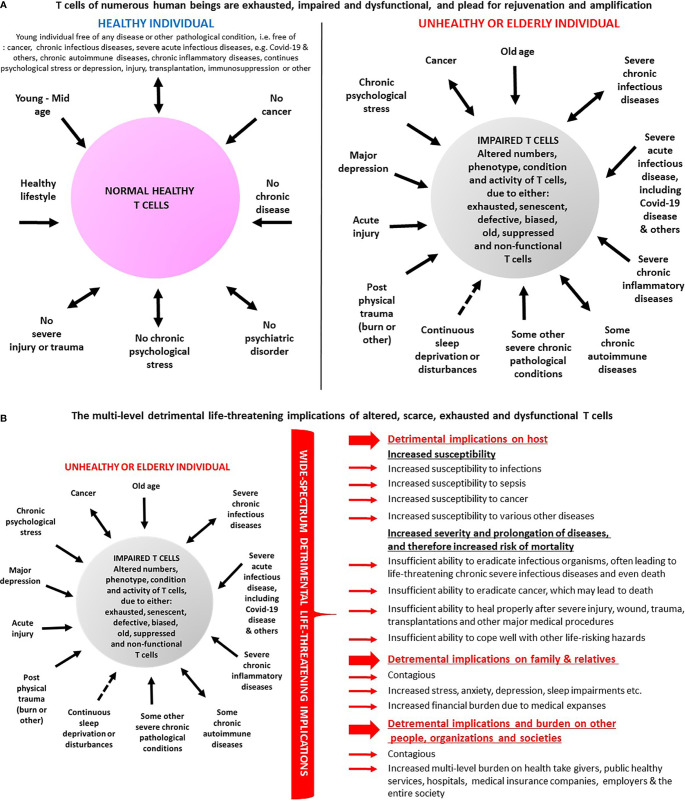
**(A)** T cells of numerous human beings are impaired and dysfunctional and ‘plead’ for rejuvenation and amplification. The panel lists several conditions in which T cells are normal and healthy and therefore do not function properly (left), *vis-à-vis* multiple conditions in which T cells are altered in various ways, *i.e*. decreased in their numbers, and/or exhausted, senescent, impaired, defective, biased, and dysfunctional (right). Some of the multiple published evidences for these T cell alterations in human beings are cited in the paper. **(B)** Some of the envisioned multi-level detrimental implications of altered, scarce, exhausted, senescent and dysfunctional T cells. The various different T cell impairments, in people in a kaleidoscope of abnormal conditions (left), do not allow effective eradication of infectious organisms and of cancer cells, and prevent, limit or impair other absolutely essential T cells functions in the periphery and in the brain. Some of the negative consequences and implications of these T cell impairments (right) are the following: **1**. The people whose T cells are suboptimal in number and function are more susceptible to infectious organisms: viruses, bacteria, fungi, and parasites, and cannot eradicate them in an optimal manner. **2**. People carrying infectious organisms for prolonged periods, on top of suffering themselves from chronic infectious diseases, also become contagious and can infect many other people. This high risk creates a need of, and leads to and a very difficult and problematic mission of, social distancing and even complete isolation of the infected people as seen in the SARS-Cov-2 (Covid-19) pandemics. **3**. The T cell impairments do not allow effective prevention and eradication of cancer. Needless to remind the readers that cancer kills, and that so do certain harsh complications and side effects of some anti-cancer treatments. **4**. The T cell impairments can limit, and even distort, the person’s responses to some vaccinations, drugs and medical procedures, and as such may lead to unexpected side effects, which would not occur in people with normal and properly functioning healthy T cells. Due to that, I would argue that it is very important to perform routine and periodic tests for the entire population, to analyze the T cells of each person for their total counts, as well as analyze their phenotype, subpopulations, condition, and overall functionality. Such recommended routine personalized tests may have profound impact and implications, as they could indicate if the person's T cells are in normal number and condition, or rather scarce, exhausted, senescent and dysfunctional. I further claim that such tests must become obligatory prior to any administration of vaccination, or drugs, performance of surgery, or other medical procedure. **5**. The immunocompromised people whose T cells are scarce and dysfunctional, and that become increasingly and chronically ill, often for many years, become a physical, physiological, and economical burden on hospitals, in-hospital medical staff, emergency and intensive care units, critical medical instruments, out-of-hospital healthcare providers, insurance companies, and other people and organizations. **6**. The low number and abnormal T cell function of elderly people, and of patients with various diseases, and the increased susceptibility and risk of the respective people, can induce severe ongoing/chronic stress, depression, fear and anxiety. These psychological problems can affect later also their family members and other relatives and colleagues. This increased stress, often becoming chronic, has by itself multiple very severe and well-documented detrimental effects on the health of all these people. **7**. The people whose T cells are scarce and dysfunctional, become a heavy clinical, social can physiological and economical problem of, and a heavy burden on, their society and country, due to all the above.

However, in contrast to T cells of healthy subjects (**Figure 1A** **-**left side), the T cells of numerous people in various abnormal conditions (**Figure 1A** **-**right) are scarce and/or exhausted, senescent, impaired, biased, and often also suffer from altered stemness and therefore do not function properly.

This is the case for elderly people (4–10), and for patients with either: chronic infections (11–20), acute SARS-Cov-2 infection and the resulting Covid-19 disease (21–27), cancer (2–5), chronic-stress (28–33), major depression (6, 7), sleep abnormalities (7, 8), and major injury/trauma (2, 3). This is also the case for immunosuppressed people having T cells that are suboptimal due to various other reasons among them: lymphoablation, transplantations, immunosuppressive drugs, and other procedures and conditions that weaken the immune system.

T cell exhaustion refers to deterioration of T cell function. Exhausted T cells (Tex) are characterized by low proliferation in response to antigen stimulation, progressive loss of effector function (cytokine production and killing function), expression of multiple inhibitory receptors such as PD-1, Tim3, and LAG3, and metabolic alterations from oxidative phosphorylation to glycolysis (4, 9–16). Exhausted T cells are further discussed below in the section *Severe Chronic Viral Infections*.

T cell senescence or biological aging is a process that results from a variety of stresses, and leads to a state of gradual deterioration of functional characteristics and irreversible growth arrest (4, 16–18).

T cell stemness is characterized by the capacity of T cells to self-renew, and be multipotent. T cell stemness combines the ability of a T cell to perpetuate its lineage and give rise to differentiated cells, and to interact with its environment to maintain a balance between quiescence, proliferation, and regeneration (4, 19).

Individuals who have scarce and/or exhausted, senescent, altered and dysfunctional T cells are very susceptible and at high risk of morbidity and mortality, and become a harsh multi-level clinical, physiological, and economic burden on many other people, organizations, and societies (specified only in **Figure 1B** **-**right and in further detail in its legend, due to word limit of this paper).

## T Cells of Numerous Human Beings are Impaired and Dysfunctional

### Aging

T cells undergo characteristic deterioration with age, leading to increased incidence of cancer and infectious diseases, reduced immunogenicity and efficacy of many vaccines, and problematic recovery after injuries, surgeries, and transplantations (16, 20).

The T cell impairments in elderly people are due to decreased output of lymphoid cells from the bone marrow, and the involution of the thymus (16). Concomitantly, there is an accumulation of highly differentiated T cells that previously encountered antigens, and this leads to diminished T cell receptor (TCR) repertoire.

T cells of aging people share some similarities with senescent cells (17), *i.e.* cells that underwent a permanent proliferative arrest. The senescent cells stop dividing due to various stress-inducing factors, among them both environmental and internal damaging events, abnormal cellular growth, oxidative stress, autophagy factors, and others (16, 17). The similarities between T cells of elderly people and senescent cells include shorter telomers, accumulated DNA damage, and metabolic changes (16, 17).

### Severe Viral Infections

#### Severe Chronic Viral Infections

T cell exhaustion, evident by dysfunction or physical elimination of antigen-specific T cells, occurs in various chronic infections, by human immunodeficiency virus (HIV), hepatitis B virus (HBV), hepatitis C virus (HCV), and other microorganisms, and in cancer (4, 9–16).

CD8^+^ T cells are important for protective immunity against intracellular microorganisms and tumors, but in chronic infections or cancer, the CD8^+^ T cells are exposed to persistent antigen and/or inflammatory signals, leading to a gradual deterioration of their function, a state called “exhaustion”. T*ex* are characterized by progressive loss of effector functions (cytokine production and killing function), expression of multiple inhibitory receptors (such as PD-1 and LAG3), dysregulated metabolism, poor memory recall, homeostatic self-renewal, homeostatic proliferation, and distinct transcriptional and epigenetic programs (11–20).

T*ex* are a distinct cell lineage that arise during chronic infections and cancers in animal models and humans. T*ex* cells are heterogeneous and include progenitor and terminal subsets with unique characteristics and responses to checkpoint blockade (11–20).

These altered functions are closely-related with altered transcriptional program and epigenetic landscape that clearly distinguish Tex from normal effector and memory T cells. T cell exhaustion represents a continuous spectrum of cellular dysfunction induced during chronic viral infection, facilitating viral persistence and associating with poor clinical outcome. Several types of modulations of T cell exhaustion can restore function in T*ex*, promoting viral clearance (14).

#### Severe Acute Viral Infection: SARS-Cov2 Infection (Covid-19, Coronavirus)

T cells are extremely important for immune resistance to SARS-CoV-2, and for protecting affected people from developing very severe Covid-19 disease (21, 22).

Several recent studies document dramatic reduction in the total number of T cells and specifically in CD4^+^ and CD8^+^ T cells, with functional exhaustion of T cells, in SARS-CoV-2-infected patients, especially in patients requiring intensive care (21, 22).

Counts of total T cells, CD8^+^ T cells or CD4^+^ T cells lower than 800, 300, or 400/μl, respectively, negatively correlate with survival of SARS-CoV-2-infected patients (12). T cell numbers are negatively correlated with serum IL-6, IL-10, and TNFα levels. Patients in disease resolution period show reduced levels of these cytokines and restored T cell counts. T cells from SARS-CoV-2-infected patients have significantly higher levels of PD-1 (12). Moreover, increased PD-1 and Tim-3 expression on T cells is seen as patients progress from prodromal to overtly symptomatic stages (12).

### Cancer

Many cancer patients often have low number of effector T cells (Teffs), and these cells suffer from anergy, exhaustion, senescence, and impaired stemness (4, 5, 11, 14, 15). T cell impairments in cancer patients are caused by several factors that can be grouped in several very broad categories, among them: Category 1: The adverse effects imposed on T cells by the cancer cells; Category 2: The anti-T cell deleterious side effects of various cancer treatments: chemotherapy, immunosuppressive drugs, other drugs, radiation and surgery; Category 3: Psychosocial factors including ongoing severe stress, anxiety, depression, fear, pain, sleep disturbances; Category 4: Cancer-related malnutrition.

Thus, the T cells of cancer patients are often dysfunctional and unable to eradicate the cancer cells efficiently, due to many different reasons. On top of the above-mentioned division into general categories, the reasons and factors that limit and/or impair the T cells of cancer patients can be divided into distinct families, each containing multiple specific members, as discussed in detail in (11). Among these are the following:

Factor family 1: Lack of antigen processing, T cell recognition and TCR signaling; Factors family 2: Negative immune modulation; Factors family 3: Non-accessibility of tumor to T cells; Factors family 4: Immune editing, equilibrium and escape (11). Due to their low numbers and multiple impairments, the cancer patient’s T cells are unable to eradicate effectively both the cancer cells infectious organisms that infect the patients, and are also unable to perform proper tissue repair and their other essential and beneficial tasks in the periphery and brain.

On these grounds, an effective and safe rejuvenation of scarce, exhausted and dysfunctional T cells, and induction of multiple beneficial T cell functions in cancer patients, remain an urgent desired clinical goal.

### Chronic Stress, Severe Depression, and Sleep Disturbances

Chronic stress is associated with immune dysfunction and various peripheral T cell abnormalities, including increased frequency and suppressive function of CD4^+^CD25^+^ and CD4^+^FoxP3^+^ regulatory T cells (Tregs), synergistic decreased function of effector T cells (Teffs) and antigen presenting cells (APCs), and a shift of the Th1 to Th2 cytokine responses (23). The increased percentage of Tregs is associated with inflammatory and neuroendocrine responses to acute psychological stress and poorer health status in older men and women (23).

A subset of individuals with major depressive disorder (MDD) have impaired adaptive immunity characterized by a greater vulnerability to viral infections and deficient responses to vaccinations, along with decreased number and/or activity of T cells and natural killer cells (NKCs) (7).

MDD patients also have significantly increased percentage of CD127^low^/CCR4^+^ Tregs, and memory Tregs, and reduced CD56^+^CD16^-^ (putative immunoregulatory) NKC counts (7). Moreover, CD4^+^ T cells of MDD patients are characterized by higher frequencies of CD4(+)CD25(high)CD127(low/-) cells, higher FOXP3 mRNA expression, and less diverse TCR V*β* repertoires (6). T cells from MDD patients also suffer from significantly lower surface expression of the chemokine receptors CXCR3 and CCR6, which are central to T cell differentiation and trafficking.

Sleep disturbance, which is a core symptom of MDD, is by itself associated with alterations in lymphocyte distributions. Within the MDD group, self-reported sleep disturbance was associated with an increased percentage of effector memory CD8+ cells, but with a lower percentage of CD56+CD16− NKC (7).

Moreover, sleep deprivation disturbs severely the functional circadian rhythm of natural Treg counts, and reduces CD4^+^CD25^-^ T cell proliferation (7). MDD and associated sleep disturbances also increase effector memory CD8^+^ and Treg pathways (8).

Together, these findings indicate that the T cell phenotype and TCR utilization are skewed on several levels in MDD patients and in people with sleep disturbances, and as such may increase the patient’s susceptibility to infectious diseases and cancer. It is hypothesized that these T cell abnormalities may even contribute to abnormal cognitive brain function and deficient CNS protection.

Interestingly, CD3^+^ T cells were shown to be critical for resolution of comorbid inflammatory pain and depression-like behavior in a model of peripheral inflammation (24). Indeed, it was shown that T cells were required for resolution of the comorbid persistent mechanical allodynia, spontaneous pain and depression in this model of peripheral inflammation, indicating that the immune system can contribute to both the onset and resolution of these comorbidities (24). It was suggested that pro-resolution effects of T cells may have a major impact on treating patients with comorbid persistent pain and depression (24).

### Acute Wound, Injury and Trauma

Lymphocytes are essential for wound healing and tissue repair (25). For example, in response to wound, on top of neutrophils’ first and monocytes’ later recruitment to the clot, as a first line of defense against bacteria, the adaptive immune system comprising Langerhans cells, dermal dendritic cells and T cells, are also activated to combat self and foreign antigens (25).

Serious injury in humans and in experimental animals is associated with severe decreases in T cell-dependent immune functions, leading to generalized immunosuppression, which, in turn, increases host susceptibility to infections and sepsis (26, 27).

Following severe injury, there is a diminished production of IL-12, loss of Th1 function and cytokine production, and a shift to a Th2 phenotype, with increased production of IL-4 and IL-10 cytokines known to inhibit Th1 function. Moreover, the interactions between the innate and adaptive immune systems is disturbed following injury (26).

These T cell impairments are associated with decreased resistance to infections, and can impair multiple essential T cell-mediated activities in both the peripheral organs and the nervous system. Some immunomodulatory strategies had success in animal models in ameliorating the diminished resistance to infection commonly seen after major traumatic or thermal injury (26, 27). In the review by Lederer et al., entitled *The effects of injury on the adaptive immune response*, the authors discuss the immunomodulatory strategies in animal models that succeeded in improving resistance to infection after major traumatic or thermal injury, but emphasize that immunomodulatory treatments that are successful in preventing infection may be contraindicated once infection is manifest (26).

## General Suggested Criteria for an Optimal Solution to Rejuvenate and Improve Scarce and Dysfunctional T Cells of Numerous People in a Kaleidoscope of Abnormal Conditions

The scarce, and often impaired, exhausted and dysfunctional T cells of so many people (**Figure 1A**-right, **Figure 1B**-left), and their severe multi-level consequences (**Figure 1B**-right), create an urgent global need for new out-of-the box solutions to overcome the problem.

I humbly suggest, without underestimating or ruling out any other proposed solution, that the optimal/best desired cellular method for rejuvenation and amplification of beneficial T cell functions, should hopefully meet all the following 20 criteria.

1. Be personalized,

2. Be safe and free of any detrimental side effects, and in fact lead only to positive side effects on the person’s overall well-being, owing to the improved T cell function,

3. Be as natural/physiological as possible, by using natural signaling molecules which bind and activate their natural receptors, and by doing so improve patient’s own T cells,

4. Be very effective, by inducing and improving simultaneously or sequentially multiple beneficial T cell features and functions (rather than only a single effect),

5. Be patient-friendly and painless, do not necessitate hospitalization or any other procedure or treatment, and allow continuation of everyday life during therapy,

6. Be applicable to many people in very different ages and abnormal conditions, who suffer from scarce, sub-optimal and dysfunctional T cells,

7. Allow repeated and timely therapy whenever needed, and for as long as needed, and as such allow even prolonged periodic treatment over months and years, for people with chronic T cell dysfunction,

8. Be flexible and contain several build in options and modalities, allowing higher degree of freedom and action,

9. Contain preliminary personalized ex vivo diagnostic cellular functional tests that can be done at any time, using small quantity of the patient's own T cells (purified from small quantity of peripheral blood), and reliable biomarkers. These ex vivo tests should be able to evaluate the present condition of the patient's T cells, and measure their ex vivo responsiveness to the planned adoptive cellular therapy. As such, these diagnostic tests could evaluate and predict the person’s potential benefit from tentative treatment and to tailor personalized regimen,

10. Be independent of a prerequisite of knowing *a priori* the antigen/s expressed on the disease-causing cells or microorganisms. Therefore, the method should not be limited to, or suitable only for, cases in which either the tumor antigens or the antigens of infectious organisms (e.g. the spike protein of Covid-19) are known and can be used.

11. Not depend on, and not utilize, the T cell receptor (TCR) and its associated proteins, for avoiding both further Activation-Induced Cell Death (ACID), T cell exhaustion, and the risk of autoimmunity,

12. Not manipulate the T cells genetically,

13. Not park/culture the T cells *in vitro* for prolonged periods, for not losing some essential traits/capabilities. It is especially important that the therapeutic method would not impair T cell ability to migrate and home to, and penetrate into, various organs and tissues which either contain disease-causing cells or infectious organism, or are injured and require T cell help for healing,

14. Not use T cells whose *in vivo* activity is dependent on cytokines or growth factors and need such subsequent support *in vivo*. Thus, the therapeutic method should preferably be free of any detrimental side effects of systematically-administered cytokines (e.g. IL-2), and of any cytokine storms,

15. Not perform any pre conditioning procedure prior to the therapy itself (e.g. chemotherapy or biologics based lymphoablation, radiation or others), and do inject any drug before, during, or after the infusion of the adoptive transfer of the patient's rejuvenated and improved T cells,

16. Not change, inactivate or even suppress transiently any natural receptors, ion channels, or other proteins expressed on the T cell surface during the *ex vivo* process of T cell rejuvenation, activation and amplification, prior to the inoculation of the patient's improved T cells into his body,

17. Not necessitate single infusions of billions of improved T cells each time, for avoiding *in vivo* cytokine storms and competition over natural ingredients and space. Settle with much less T cells injected repeatedly over few weeks or months

18. For conditions which are NOT cancer, use and adoptively transfer rejuvenated and improved T cells which can be “friendly” to, and communicate with, other cells, rather than very aggressive T cells which may damage, or compete with other cells for natural resources and space, in lymphoid organs, and in other tissues and organs, and which may also cause autoimmunity,

19. The new immunothreapy should stand on its own, as a mono therapy, for saving, prolonging, and improving life, but must not interrupt or compete with any other efficient treatment/drugs from which the person can benefit from, and which may overcome T cell exhaustion and allow better T cell function. Thus, any other prior, simultaneous, in between, or later safe and effective treatment would be possible, as long as they do not harm T cells and do not induce immunosuppression.

20. On top of being used on their own, it would be advantageous if the diagnostic and therapeutic methods and protocols of the new immunotherapy could be used also as "add on technologies'', and allow improvement of other adoptively-transferred therapeutic T cells.

## T Cell Immunotherapies—“The Medical Equivalent of Splitting the Atom”

T cell immunotherapies (34, 35) have revolutionized medicine and even called by The New York Times: “The medical equivalent of splitting the atom” (28).

In line with this scientific and clinical revolution, there is an enormous number of scientists and clinicians working on T cell immunotherapies, and a meteoric rise of companies developing and utilizing them, primarily for cancer, but also for some infectious diseases. Current adoptive/cellular T cell therapies include mainly the following types: donor lymphocyte infusions, tumor-infiltrating lymphocytes, T-cell receptor-engineered T cells, chimeric antigen receptor (CAR) T cells, and virus-specific T cells. These T cell immunotherapies are reviewed in many papers, among them (34, 35).

While each of these potent T cell immunotherapies has their own clear focus, advantages, and successes, primarily for some types of cancer, to the best of my knowledge none can rejuvenate and rescue T cells from exhaustion and senescence whenever needed and for whoever needs it, and that manage to improve multiple T cell functions simultaneously, among them increase migration, homing and extravasation into tissues (absolutely essential for penetrating and combating “Cold tumors”).

I’m also not aware of adoptive T cell immunotherapies that fulfil all, or at least most, of the 20 suggested criteria specified in the preceding section, and are suitable for broad use for all, at least most conditions of scarce, exhausted, suboptimal, and dysfunctional T cells.

## Hypothesis and Suggestion: “Nerve-Driven Immunity” May Rejuvenate, Activate, and Amplify Beneficial T Cells in a Direct, Safe, and Effective Manner

How can we safely and potently rejuvenate, activate, and improve scarce, exhausted and/or senescent T cells whenever needed and fulfil the 20 suggested criteria for optimal solution defined in the previous section?

**Figure 2:**
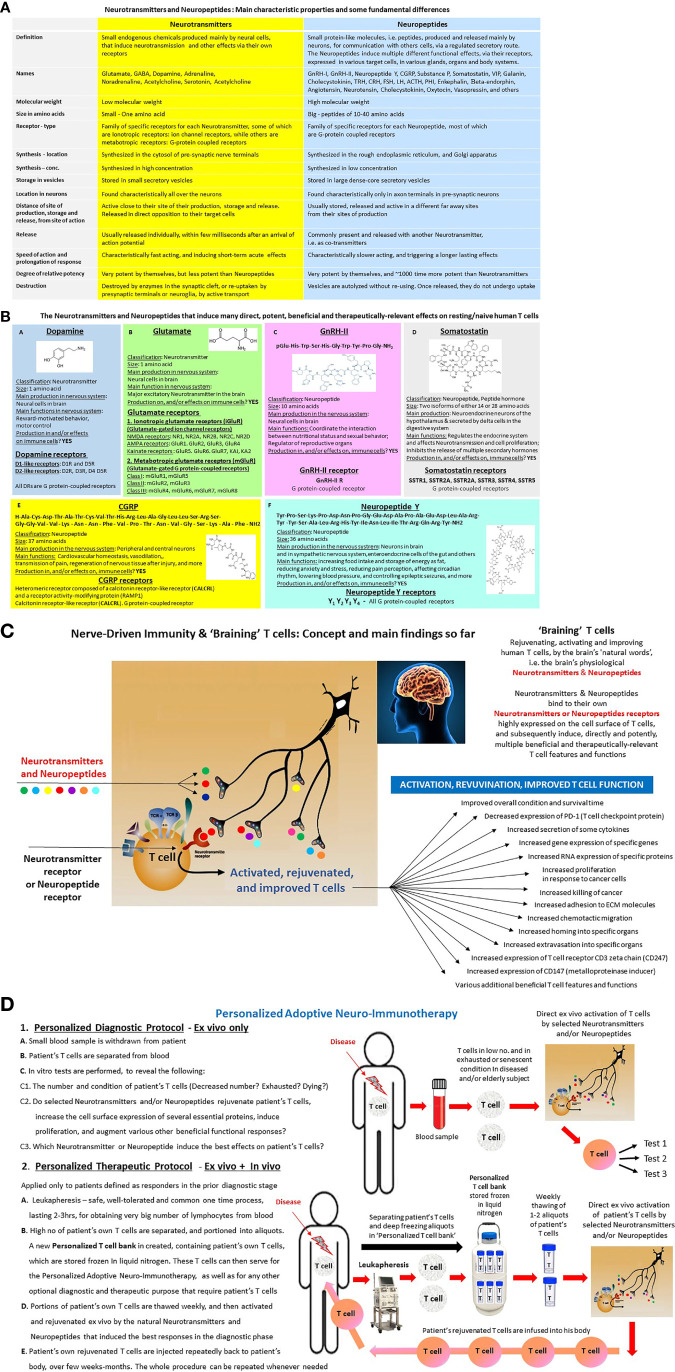
**(A)** Neurotransmitters and Neuropeptides—Definitions and characteristic features in the nervous system. Neurotransmitters are traditionally defined as endogenous chemical substances used by the nervous system to transmit messages either between neurons, or from neurons to muscles, or from neurons to gland cells. The communication between two neurons happens in the synaptic cleft (the small gap between the synapses of neurons), where electrical signals that have travelled along the axon are briefly converted into chemical ones through the release of Neurotransmitters, causing a specific response in the receiving neuron. A Neurotransmitter influences a neuron in one of three ways: excitatory, inhibitory or modulatory. An excitatory Neurotransmitter promotes the generation of an electrical signal called an action potential in the receiving neuron, while an inhibitory Neurotransmitter prevents it. Whether a Neurotransmitter is excitatory or inhibitory depends on the receptor it binds to. Neuropeptides are traditionally defined as small protein-like molecules, *i.e.* peptides, produced and released by neurons through the regulated secretory route, and acting on neural substrates and additional ones. The Neuropeptides are derived from precursor molecules that must be post-translationally processed to yield the active peptides. The Neuropeptides may diffuse for long distances within the extracellular space before binding to their specific receptors, which are almost exclusively G protein-coupled receptors. The Neuropeptides and their receptors modulate many diverse functions of the central nervous system, including sleep, arousal, reward, feeding, pain, cognition, stress responses, and emotions. **(B)** Basic features of few Neurotransmitters and Neuropeptides that induce many direct, potent, beneficial and therapeutically-relevant effects on resting/naive human T cells. The Fig includes basic information about Dopamine, Glutamate, GnRH-II, Somatostatin, CGRP and Neuropeptide Y, and about their receptors. These Neurotransmitters and Neuropeptides induce very potent effects on various “classical” well-known target cells throughout the body. These Neurotransmitters and Neuropeptides induce multiple direct, potent, beneficial and therapeutically-relevant effects in resting/naive human T cells, and in some of other immune cells. Each of these Neurotransmitters and Neuropeptides has a family of its own receptors, expressed in different levels and compositions in its target cells, T cells included. **(C)** Nerve-Driven Immunity & ‘Braining’ T cells: Concept and main findings so far. The figure shows the principle elements of ‘Nerve-Driven Immunity’ (30, 31), allowing direct communication between the brain and T cells, via Neurotransmitters and Neuropeptides secreted by nerve endings, and their receptors expressed on the cell surface of T cells. The figure also shows a partial list of the direct effects that some Neurotransmitters and Neuropeptides, primarily: Dopamine, Glutamate, GnRH-I, GnRH-II, CGRP, Somatostatin and Neuropeptides Y, induce in resting/naive human T cells, revealed in our experiments so far. Most of these effects are described in our published papers (29–33, 36–45), but some are still unpublished data, or submitted to publication. **(D)** Personalized Adoptive Neuro-Immunotherapy. This is a new mode of personalized adoptive/cellular immunotherapy, that was developed on the basis of the direct, rapid, potent and beneficial effects that selected Neurotransmitters and Neuropeptides induce on their own in resting/naive human T cells (29–33, 36–45). The aim and vision of the Personalized Adoptive Neuro-Immunotherapy is to rejuvenate, activate and improve scarce, exhausted, senescent, impaired and dysfunctional T cells, in any person having such T cells, and which is in need of immunotherapy. The Personalized Adoptive Neuro-Immunotherapy was designed to meet all the 20 criteria specified in the text of this paper. The Personalized Adoptive Neuro-Immunotherapy has two stages and corresponding protocols: Personalized diagnostic protocol—*Ex vivo* only, and Personalized therapeutic protocol—*Ex vivo + In vivo*. The Personalized Adoptive Neuro-Immunotherapy diagnostic protocol: consists of few parallel *in vitro* tests, performed in parallel, on a small number of the person's own T cells, soon after their separation from his small blood sample. These tests can be performed for any human individual, at any time, and repeated at any stage. Person’s T cells are separated from a small quantity of blood and subsequently tested *in vitro*, during few days only, for their: **(A)**. Total number, and also number of few T cell subsets, **(B)**. Viability and overall condition, **(C)**. Beneficial functional responses to selected Neurotransmitters and Neuropeptides. (single exposure). The functional responses are judged simultaneously in several tests, which measure the levels of few well-defined T cells features and functional responses (mainly some of those we tested successfully already, and listed in **Figure 2C**, and which serve as our well-defined reliable biomarkers for T cell activation, rejuvenation, and improvement. At the end of the diagnostic phase, valuable results are obtained, and personalized decisions are being made, with regards to the chances that the given patient, at that specific time point of his life and health condition, would benefit from the Personalized Adoptive Neuro-Immunotherapy. The results of the diagnostic tests also teach which Neurotransmitter and/or Neuropeptide induce the best effects in the patient’s own T cells. Of note, we already performed successfully such diagnostic tests, with either resting/naïve normal human T cells of healthy subjects, or scarce and abnormal T cells of small number of cancer patients. The Personalized Adoptive Neuro-Immunotherapy therapeutic regimen will be administered only to patients whose T cells were found to be responsive in the pre-clinical diagnostic tests, and therefore viewed as people that can benefit from this treatment, at this specific time point of their life. The therapeutic procedure will take place weekly, for several months, and the entire therapeutic package can be repeated whenever, and for as long as, needed. During this anticipated treatment, the candidates for the therapeutic procedure will first undergo leukophoresis, and then their T cells will be separated and frozen in aliquots. By doing so, a unique and very valuable personalized T cell bank is created, that can be used both for the Personalized Adoptive Neuro-Immunotherapy, and for various other diagnostic or therapeutic purposes. Then, one to two times once or twice a week, portions of patient’s own T cells are thawed, activated, “rejuvenated”, and improved *ex vivo* by single exposure to selected natural Neurotransmitters and Neuropeptides (those found to be the best in the diagnostic phase), and inoculated soon afterwards into the patient’s body. It is envisioned that the people receiving the Personalized Adoptive Neuro-Immunotherapy will not be hospitalized, and will not need any additional treatment before, during, or afterwards. The person’s own T cells, that were rejuvenated and activated *ex vivo* by the Neurotransmitters and Neuropeptides, are expected to have substantially improved abilities to reach and eradicate cancer and infectious organisms in his body, as well as to perform all their other essential T cell tasks. By inducing all these effects, it is envisioned and hoped that the “Personalized adoptive Neuro-Immunotherapy” will improve significantly the patient’s condition in various ways and levels, and even save patient’s life. Yet, words or cautions and modesty are absolutely required here, since the therapeutic protocol has not yet been tested in clinical trials, and is of course not an approved therapy yet. The Personalize Adoptive Neuro-Immunotherapy was invented and patented by the author of this paper: Dr. Mia Levite, Israel. Currently, when the technology is IP protected, attempts are being made for bringing it closer to the patient’s bedside. A final important note, I think that repeated injection of Neurotransmitters and Neuropeptides into the patient’s body can not replace the cellular/adoptive therapy, primarily since injected Neurotransmitters and Neuropeptides will most probably induce various detrimental side effects. Thus, I fear that if a person would get repeated continuous/prolonged infusion, over few weeks or months, of either of the Neurotransmitters and Neuropeptides we used so far to rejuvenate, improve and activate T cells, these Neurotransmitters and Neuropeptides would bind their respective receptors in various cells that express their receptors, and subsequently induce various side effects within multiple organs and tissues. Such side effects would of course not happen if only T cells are exposed to Neurotransmitters and Neuropeptides, and if this exposure is only *ex vivo*, as in the suggested Personalized Adoptive Neuro-Immunotherapy.

I humbly propose, based on multiple supporting evidence so far (46–59), many of which are specified below in the next chapters, in the cited papers, and in **Figure 2C**, that this may be achievable by using *ex vivo* natural Neurotransmitters and Neuropeptides (**Figures 2A, B**), by the “*Nerve-Driven Immunity*” language (36, 37, 46), (**Figure 2C**) and by the novel *Personalized Adoptive Neuro-Immunotherapy* (**Figure 2D**).

My idea and suggestion are to try to mimic and translate into therapeutic terms, what I hypothesize that the nervous system most probably normally does in everyday life: *i.e.* “talks” directly to T cells in various parts of the body *via* Neurotransmitters and Neuropeptides, that in turn bind to their specific receptors in T cells (as well as many others cells) and induce on their own multiple direct, rapid, potent, timely and beneficial effects. I further hypothesize that T cells need Neurotransmitters, Neuropeptides and their receptors, and the direct, rapid and potent effects they induce, for performing multiple health-guarding T cell tasks, and for communicating with the brain and with other body systems and organs.

## Neurotransmitters and Neuropeptides

### Definitions and Characteristic Features of Neurotransmitters and Neuropeptides in the Nervous System

Neurotransmitters are traditionally defined as endogenous chemical substances used by the nervous system to transmit messages, either between neurons, or from neurons to muscles, or from neurons to gland cells. In addition, many Neurotransmitters induce direct effects on T cells and other immune cells, as well as on different cell types which express their receptors.

Neuropeptides are traditionally defined as small protein-like molecules, *i.e.* peptides, produced and released by neurons through the regulated secretory route, and acting on neural substrates. Once again, T cells and other immune cells ought to be added as target cells that are affected directly by Neuropeptides.

**Figure 2A** summarizes the classical features of Neurotransmitters and Neuropeptides in the nervous system. Yet, these definitions and characteristics ignore completely the direct and very potent effects of many Neurotransmitters and Neuropeptides on T cells and other immune cells, each *via* its own functional Neurotransmitter/Neuropeptide receptors that are highly expressed in these cells (29–33).

### Many Neurotransmitters and Neuropeptides Are Secreted From Both Neural and Immune Sources, and Can Reach and Affect T Cells That Express Their Specific Receptors in Both a Paracrine and Autocrine Manner, in Most/All Body Organs

Most organs are innervated, and the nerve endings secrete Neurotransmitters and Neuropeptides. The innervated body organs include: the brain, muscle, spinal cord, skin, gut, blood vessels, all the lymphoid organs and tissues (47–50), and almost all other body organs.

Interestingly, with regard to the gut- the gut microbiota has been shown to produce mammalian Neurotransmitters and Neuropeptides and/or consume them (51–53). It was also shown that Neuropeptides and Neurotransmitters contribute to the mutual microbiota–host interactions (52, 53). Thus, I envision that the microbially-derived Neurotransmitters and Neuropeptides (51) can also bind and affect some T cells in a direct and powerful manner.

T cells and other immune cells express on their cell surface functional and important receptors of most, if not all, types of Neurotransmitters and Neuropeptides, among them Dopamine receptors (33, 37, 38, 54–56), Glutamate receptors (36, 39, 40, 46, 57–61), Acetylcholine receptors (62, 63), GABA receptors (64) (65), VIP receptors (66) and others. I would therefore propose that most/all the Neurotransmitters and Neuropeptides secreted from nerve endings at the vicinity of T cells, can affect the T cells that express their receptors, in most if not all body locations in which they reside and/or patrol through, in a direct, safe, and potent manner (30–32).

In addition to the neural production of Neurotransmitters and Neuropeptides, T cells, as well as other immune cells can produce and secrete on their own some Neurotransmitters, including Dopamine and other catecholamines (67–70), Glutamate (71, 72), Acetylcholine (62, 63, 73) and several others.

Moreover, T cell-derived Neurotransmitters can affect neural cells, as shown for example for Acetylcholine-synthesizing T cells that relay neural signals in a vagus nerve circuit (73) and induce other potent effects on other cells (62, 63). Two other examples are: 1. T cell-derived glutamate that endows astrocytes with a neuroprotective phenotype (71), and 2. Glutamate produced and secreted to the extracellular milieu by stimulated Th17 cells, using a vesicular release pathway mediated by β1 integrins and Kv1.3 channel signaling, and involving SNARE protein machinery complex (72).

Furthermore, immune-derived Neurotransmitters have strong autocrine and paracrine effects on T cells, as demonstrated for example for catecholamines (70, 74, 75).

Taken together, I hypothesize that Neurotransmitters and Neuropeptides can convey rapid, potent and precise functional “messages” in at least four different routes:

1. Neuro —> Neuro route: Within the nervous system;
2. Neuro —> Immune route: From the nervous system to the immune system;
3. Immune —> Neuro route: From the immune system to the nervous system;
4. Immune —> Immune route: Within the immune system.

Neurotransmitters and Neuropeptides should therefore be considered as “NeuroImmunotransmitters” and “NeuroImmunopeptides.”

To these four routes, few additional ones can be added, for example the microbiota-host route (52, 53).

Interestingly, some of the disease conditions described in the previous parts of this paper are associated with changes in the levels and/or function of certain Neurotransmitters and/or Neuropeptides, or of their receptors. Yet, to the best of my knowledge, in many cases, the direct consequences of these changes on immune cells were not studied systematically so far. One exception is the consequences of alterations in the dopaminergic system. Curious readers may read several publications on this topic written by different authors, including my book chapter entitled: *Dopamine in the Immune System: Dopamine Receptors in Immune Cells, Potent Effects, Endogenous Production and Involvement in Immune and Neuropsychiatric Diseases* (37), and my later review on Dopamine and T cells (38). The readers are also referred to the other chapters in the book entitled: *Nerve-Driven Immunity: Neurotransmitters and Neuropeptides in the Immune system*, each chapter dedicated to a different Neurotransmitter or Neuropeptide (32).

## Nerve-Driven Immunity

### Summary of Most of Our Nerve-Driven Immunity Findings So Far

In our own multiple studies performed over the years, we found that few natural Neurotransmitters and Neuropeptides: Glutamate, Dopamine, GnRH-I, GnRH-II, Somatostatin, CGRP and Neuropeptide Y (**Figure 2B**), each by itself, at low physiological concentrations of ~10^−8^M (10 nM), induce multiple direct and potent beneficial effects on resting/naïve T cells (29–33, 36–45).

The T cells which we tested so far and found such effects include: A. Normal resting/naive (*i.e.* as is) CD3^+^ T cells of healthy subjects purified from their blood, which are in normal number and condition (36, 41–44, 56) B. CD3^+^ T cells of few patients with Head and Neck cancer - Head and Neck Squamous Cell Carcinoma (HNSCC) (45) and of few liver cancer patients. Hepatocellular carcinoma (HCC) (submitted paper) purified from their blood, which are in abnormally low number and granular condition, C. Mouse resting/naive Th0, Th1, and Th2 antigen-specific cell lines and clones (29).

In particular, we discovered that Dopamine, Glutamate, GnRH-II, CGRP, Neuropeptide Y and Somatostatin (**Figure 2B**) can induce, each by itself some, most or all the following effects: 1. Very rapid shifts of the T cell’s membrane potential (56) and * see footnotes, 2. Unique *de novo* gene expression (43) *, 3. Cytokine secretion (both “classical” and non-classical) (29, 42), 4. Integrin-mediated adhesion to fibronectin and/or laminin (36, 41–44), 5. Chemotactic migration (36, 43, 45), 6. Migration of few HNSCC patient’s T cells towards chemokines, and more important—towards their own surgically removed tumor (45), 7. Augmented T cell extravasation into certain solid organs *in vivo* (43), 8. Faster homing *in vivo* of antigen-specific T cells to cancer-bearing organs *, 9. Increase CD3zeta** expression (45), 10. increase of CD147*** expression (43), 11. Decrease significantly and rapidly PD-1 **** expression in human T cells, 12. Increase the *in vitro* T cell proliferative response to human cancer cells, 13. Increase killing *in vitro* of human cancer cells by human T cells.

Together, by inducing all these direct and beneficial effects (*i.e.* not by a single effect/mechanism/pathway), evident at various time points after their binding to their receptors in T cells, the above-mentioned Neurotransmitters and Neuropeptides can on their own improve dramatically many essential T cell functions.

Having said that, a word of caution is noteworthy: inter-individual variability, defined as intrinsic differences between people and evident with regard to responses or sensitivity to almost factor/drug or procedure, is also seen with regard to the effects of Neurotransmitters and Neuropeptides on T cells. I envision that such variabilities may be due to inter-individual variabilities with regard to the expression level and functional status of the receptors for Neurotransmitters and Neuropeptides expressed in people’s T cells, due to to their present physiological, physical, psychological or pathological condition, or even due to their nutrition or medication they take.

*Footnotes*

* *Our solid yet still unpublished data, research ongoing and new papers either already submitted or in preparation*.

**CD3zeta is a TCR-associated chain essential for eradication of cancer, but downregulated pathologically in many patients with multiple cancers. Therefore, elevating CD3zeta in T cells of cancer patients is a therapeutic goal.

***CD147 is an extracellular matrix metalloproteinase inducer, needed for cell penetration and extravasation of solid organs. Therefore, elevating CD147 in T cells can augment T cell extravasation.

****PD-1 is a physiological and beneficial checkpoint protein, a type of “off switch” protein, that delivers important negative immunoregulatory signals to T cells, to remain silent when T cell activation is not needed, and to avoid autoimmunity. However, cancer cells use T cell’s PD-1 for their own harmful purposes. Indeed, cancer cells have their PD-1 ligand (PD-1L) that bind to the T cell’s PD-1 and by doing so keep T cells in an inactive/suppressed mode, and prevent T cell reactivity against themselves. Monoclonal antibodies that target either PD-1 or PD-L1 can block this binding and boost the immune response against cancer cells. These drugs have shown a great deal of promise and success in treating certain cancers.

### Our Most Recent Findings: Neurotransmitters and Neuropeptides Decrease PD-1 in T Cells of Both Healthy Subjects and Elderly Liver Cancer Patients, and Induce Their Rejuvenation, Proliferation and Killing of Liver Cancer Cells

In our most recent study (submitted paper) we studied the direct effects of Dopamine, Glutamate, GnRH-II, CGRP or Neuropeptide Y (**Figure 2B**) on CD3^+^ peripheral T cells of few elderly people aged 79–86 years, suffering from HCC and a kaleidoscope of comorbid conditions. We also tested the effects of these Neurotransmitters and Neuropeptides on CD3^+^ naïve/resting T cells of additional healthy subjects. In all cases, the CD3^+^ T cells were purified from small blood samples.

We found all the following significant findings: 1. The HCC patients had 5–10 fold less T cells than healthy subjects, 2. The patient’s T cells were abnormal, *i.e.* very small and granular, 3. The human T cells, express all dopamine receptors: DR1-5, and glutamate receptors: AMPA-R and NMDA-R, 4. Dopamine, Glutamate, GnRH-II, Neuropeptide Y, and CGRP (each at low conc. of 10^−8^ M) induced the following effects: A. Decreased significantly both the percentage of PD-1+ T cells and the level of PD-1 expression per cell (up to 60% decrease, within in 1 hr. only), B. Increased, up to seven fold, the number of alive T cells that proliferated *in vitro* in response to human HCC cells (either HepG2 or Huh7 cell line), C. Increased significantly killing of human HCC cells *in vitro* by the T cells (up to 2 fold increase).

Moreover, we found that few unexpected combinations of Neurotransmitters and Neuropeptides induced even stronger effects than the single Neurotransmitters/Neuropeptides, and that Dopamine D1-5R agonists, of which D4R was the best, also decreased PD-1 and increased T cell numbers.

Together, these findings demonstrate that Dopamine, Glutamate, GnRH-II, Neuropeptide Y and CGRP, each on its own or in combinations, can activate, rejuvenate, and improve T cells, even when they are scarce and suboptimal T cells of elderly people with cancer and several diseases, and that they do so in low physiological concentration, single exposure and direct manner—*via* their own receptors in T cells, and by inducing multiple beneficial effects. Yet, once again, we found significant inter-individual variability with regard to the effects of these Neurotransmitters and Neuropeptides on human T cells.

## From the Bench to the Bedside: Translating “Nerve-Driven Immunity” Into “Personalized Adoptive Neuro-Immunotherapy”

Based on the all the experimental findings in hand so far and the corresponding publications (29–33, 36–45), and also on some still unpublished data, I designed a novel type of adoptive T cell immunotherapy named “*Personalized Adoptive Neuro-Immunotherapy*”, drawn schematically in **Figure 2D**, and explained in detail only in its legend (due to word limit of this paper).

I propose that the “Personalized Adoptive Neuro-Immunotherapy” has the ability to rejuvenate, activate and improve T cell number, condition, migration and function. By doing so, this novel type of adoptive cellular therapy may hopefully increase survival, improve life quality, and prevent multiple harsh multi-level implications (**Figure 1B**). I hypothesize that all these could be gained due to the ability of selected natural Neurotransmitter and Neuropeptides to decrease PD-1 in human T cells, and to increase all/most of the following beneficial T cell features and functions:

T adhesion to extracellular matrix glycoproteins, chemotactic migration, homing, extravasation into solid organs, gene expression, cytokine secretion, expression of key proteins (*e.g.* CD3zeta, CD147 metalloproteinase inducer, laminin receptor and others), proliferative response to cancer, killing of cancer, ability to recruit other immune cells to the site of disease or injury, and most probably additional advantageous effects not revealed yet.

The *Personalized Adoptive Neuro-immunotherapy* is composed of two stages and respective protocols (**Figure 2D**):

*1. Personalized diagnostic protocol*: *In vitro* evaluation of the overall condition of the candidate patient’s T cells, and the performance of several parallel quantitative *in vitro* tests, to measure the functional responsiveness of the patient’s T cells to several Neurotransmitters and/or Neuropeptides. These diagnostic tests aim to reveal if the patient's T cells, at that specific time point and present healthy condition, respond favorably to the Neurotransmitters and Neuropeptides, and if indeed so, to select the best Neurotransmitter and/or Neuropeptide for activating and improving these T cells. This protocol, using relatively small number of patient's own T cells, soon after their purification from his blood sample, can be applied to anyone, at any time. We already performed successfully such personalized diagnostic tests on T cells of many healthy people, few HNSSC patients and few HCC patients, and received very good and encouraging results [(38, 40, 54–64), and new submitted paper];

*2. Personalized therapeutic protocol:* The actual Neuro-immunotherapy, applied only to patients whose T cells passed successfully the first pre-clinical diagnostic tests.

The Personalized Neuro-immunotherapy is designed to meet all the 20 criteria specified herein, in one of the preceding chapters, and be safe, effective, painless, patient-friendly, and importantly, without requiring hospitalization. However, it should be emphasized that this therapeutic protocol was not tested yet in clinical trials, and of course not approved yet for clinical use.

In principle, the “Personalized Adoptive Neuro-immunotherapy” may be applied to all people with scarce, exhausted, and suboptimal T cells (**Figure 1A**-right).

Having said that, I’m fully aware of the realistic possibility that this Neuro-Immunotherapy will not be applicable to, or sufficiently effective and beneficial for, all people, in all conditions. Only clinical trials can teach us who are the human beings that benefit from it the most.

Currently, the “Personalized Adoptive Neuro-immunotherapy” is IP protected, and various actions are performed for further testing its safety and efficacy, and for bringing it closer to the patient’s bedside.

## Concluding Reflections: Neurotransmitters and Neuropeptides Talk Directly and Beneficially With T Cells, and All Sides Can Gain

My opinion and vision is that the direct communication between the nervous system and T cells and other immune cells, *via* Neurotransmitters and Neuropeptides (on top of *via* other signaling molecules), is essential and beneficial for all involved sides, and can be translated into therapeutic terms (**Figure 2D**).

Healthy ongoing bi-directional communication between the nervous system and the immune system (76), and T cells being activated and improved by Neurotransmitters and Neuropeptides, are expected to contribute substantially to a wide range of essential health-guarding T cell activities in peripheral organs, to better function and protection of the brain (2, 3, 76), and even to better resolution of comorbid persistent stress, depression, and pain (24).

In fact, I envision that all sides and parties can gain and benefit from direct activation of T cells by Neurotransmitters and Neuropeptides in multiple aspects and levels, as specified in the coming sentences.

### What Could the T Cells and the Entire Immune System Gain?

1. An ability for T cells to be activated and respond very fast, even within seconds (56), a feature not afforded by “classical” immunological signals;

2. Direct, accurate, real-time, and beneficial T cell activation, which is dictated by the brain and other parts of the nervous system, in a coordinated and orchestrated manner, and which takes place whenever needed, and wherever needed (*i.e.* in any needed body location), according to the needs of the entire body either in health or disease;

3. Rapid, transient and advantageous T cell activation *via* natural Neurotransmitters and Neuropeptides and their respective receptors expressed in T cells, which can take place independent of the TCR and specific foreign antigens. Thus, the activation of T cells *via* Neurotransmitters and Neuropeptides is not antigen-specific, and as such it can be safer, broader, and maybe even better in few contexts, since it is not expected cause neither TCR-mediated autoimmunity against self antigens, nor TCR-mediated T cell exhaustion upon repeated exposure to tumor antigens or infectious antigens.

### What Could the Nervous System Gain?

Whole body control, orchestration, coordination and adaptation, allowable by the direct real-time information conveyed to, and received from, T cells and other immune cells, in either health or disease.

### What Could Medicine Gain?

I humbly propose that we may have the ability to protect, improve, and even save many people’s lives, by using and actually mimicking the natural “language” by which the brain “talks” to T cells, for new and potentially very safe and effective mode of therapy—The “Personalized Adoptive Neuro-Immunotherapy” (**Figure 2D**). This Neuro-Immunotherapy may be beneficial for a wide spectrum of very different pathological conditions in which T cells are scarce, exhausted, impaired and dysfunctional.

### Who Could Gain?

If the “Personalized Adoptive Neuro-Immunotherapy” turns out to be indeed safe, effective and patient-friendly, an enormous number of people whose T cells are malfunctioning, as well as their relatives, health caregivers, healthy services, hospitals, insurance companies, and entire societies, could benefit from it (**Figure 1B**).

Moreover, I foresee that repeated periodic strengthening of T cells of people at risk, especially in older age, can become almost routine, generic, and broad spectrum method of immune-protection, not limited each year only to few selected microbial organisms. The current Covid-19 pandemics teach all of us a devastating and warning lesson: relying only on vaccinations to already identified viruses and other infectious organisms, is not sufficient, and can lead to disastrous worldwide implications.

## Data Availability Statement

The original contributions presented in the study are included in the article. Further inquiries can be directed to the corresponding author.

## Author Contributions

The author confirms being the sole contributor of this work and has approved it for publication.

## Conflict of Interest

The author declares that the research was conducted in the absence of any commercial or financial relationships that could be construed as a potential conflict of interest.
